# Potential causes of subfertility in patients with intramural fibroids

**DOI:** 10.1186/s40738-015-0005-2

**Published:** 2015-08-25

**Authors:** Bruce D. Pier, G. Wright Bates

**Affiliations:** Division of Reproductive Endocrinology and Infertility, Department of Obstetrics and Gynecology, The University of Alabama at Birmingham, WIC 10390, 1700 6th Avenue South, Birmingham, AL 35294 UK

**Keywords:** Leiomyoma, Fibroids, Implantation, Uterine peristalsis, Pseudocapsule

## Abstract

**Background:**

Intramural leiomyomas have been long debated as a potential cause of infertility and pregnancy loss.

**Findings:**

Previous research has linked intramural fibroids to defective implantation, as well as to abnormal peristaltic events of the uterine smooth muscle. Previous reports describe the effects of intramural fibroids on normal human fertility and early pregnancy loss, specifically in regards to implantation failure.

**Conclusion:**

A thorough understanding of prior research may direct new research focus, leading to better understanding of leiomyoma-associated infertility.

## Introduction

Uterine leiomyomas, or fibroids, can occur in up to 60 % of women before the age of 40, and 80 % of women before the age of 50 [1]. Fibroids may be the sole cause of infertility in 2–3 % of women [2]. Studies suggest an association between the presence of submucosal fibroids and subfertility and pregnancy loss, however the reproductive sequelae of intramural fibroids are even less clear [3]. Some studies have shown no association between intramural (IM) fibroids and in vitro fertilization (IVF) success [4, 5], while others have indicated the presence of IM fibroids reduces the success of IVF cycles [6, 7]. A meta-analysis of 19 observational studies suggests that non-cavitary-distorting IM fibroids have a significantly reduced clinical pregnancy rate and live birth rate (RR of 0.85, and 0.79, respectively) in IVF cycles [8]. However, many of the early studies assessing the effect the IM fibroids on fertility do not stratify by fibroid size or location, proximity of fibroids to the endometrium, age of the patient at time of diagnosis, or the method the fibroid was detected. These factors limit the applicability of these studies to clinical practice. A recent study delineated fibroids into two categories: fibroids smaller and larger than 2.85 cm, and found that the effect of fibroids impairing delivery rate after IVF did not become significant until fibroids reached a size greater than 2.85 cm [9]. While this study does address myoma size, it fails to mention proximity of the fibroids to the endometrial cavity. The Federation Internationale de Gynecologie et d’Obstetrique (FIGO) released a leiomyoma classification system to assist in classifying and treating abnormal uterine bleeding [10]. It is not clear if this classification system will assist in a better understanding of the effects intramural fibroids have on reproduction and pregnancy loss. The disparity in the current body of research illustrates the need to better characterize the impact of fibroid size, location and number on reproductive health.

Uterine fibroids have distinct gross and microscopic morphologic findings that distinguish them from malignancies. Several subtypes of leiomyomas exist, as shown in Table 1. The varying gross and morphologic presentation of uterine fibroids (Table 1) demonstrate the limited understanding about the effects of this variable benign tumor on human fertility. One such fibroid subtype, leiomyomas with bizarre nuclei (LBNs), has recently been linked to higher levels of a regulator of apoptosis, MIB-1 (mindbomb E3 ubiquitin protein ligase 1, also referred to as Ki-67) in the endometrium [11]. MIB-1 is also elevated in endometriosis [12]. This finding may suggest that not just fibroid size or location, but even fibroid morphologic subtype may play a role in subfertility seen in patients with uterine fibroids. There is currently no evidence that imaging can accurately identify morphologic subtypes, which makes diagnosing fibroid morphology subtypes during pregnancy not possible. Further research may correlate leiomyoma microscopic subtypes with different effects on human fertility.

**Table 1:** Leiomyoma subtypes and associated microscopic findings

Benign leiomyoma subtype	Microscopic findings
Benign leiomyoma	Findings include fascicles of spindle cells mixed with varying levels of collagen. Large blood vessels are present, and there is mild to absent cellular atypia and mitosis [58].
Mitotically active leiomyoma	Fibroid findings include necrosis, hemorrhage, vascular intrusion. Contains 5–9 mitotic figures (MF) per 10 high power fields (HPF) with no atypia [59].
Leiomyomas with bizarre nuclei (LBNs)	Microscopically, LBNs have the presence of aytipical pleomorphic nuclei. They may have high cellularity, and contain 2–7 MF/10HPFs [60].
Hydropic leiomyoma	These fibroids have focal collections of edema-like fluid and show hyaline degeneration [61].
Myxoid leiomyoma	Findings include large collections of acellular myxoid matrix, rich in acid mucins. They demonstrate a low mitotic rate (<2 MF/10 HPFs) [58].
Epitheloid leiomyoma	These benign leiomyomas have at least 50 % of cells as epitheloid, but demonstrate a low mitotic rate (<3MFs/10HPFs) [58].

Surgical removal of fibroids via myomectomy has been long purported to improve fertility in patients with infertility secondary to myomas [13, 14]. While there may be a small protective benefit of myomectomy for spontaneous abortion, the evidence to date does not suggest that myomectomy for IM fibroids improves pregnancy rates and ongoing live birth rates [15, 16]. There are a limited number of well controlled studies to address the efficacy of fibroid removal and as mentioned above, additional research is needed to address the impact of fibroid size, number, and proximity to the endometrium in intervention trials as well [17]. While reviews have previously addressed potential pathophysiological implications of uterine fibroids, none have focused on the pathophysiological effects IM fibroids may have on fertility and pregnancy loss [18]. The goal of this manuscript is to provide as a narrative review of the literature for potential effects that uterine fibroids may have on normal fertility, with specific attention to intramural myomas. Information included in this review was obtained via PubMed searches for articles published in the English language from the year 1960 to 2014. The following key words were used in to reveal articles of relevance: “leiomyoma”, “myoma”, “fibroid”, implantation”, “peristalsis”, “IVF”, in vitro fertilization”, “fecundity”, “myomectomy”, and well as the titles of known endometrial markers of implantation. Case reports or descriptions of therapies without information regarding evaluations of fertility were not included in this review. The potential effects of intramural fibroids were separated into alterations in implantation factors, alterations in the uterine junctional zones, effects of the fibroid pseudocapsule, and abnormal uterine peristalsis.

## Findings

### Alterations in implantation factors

HOXA10 is a widely studied homeobox gene which is responsible for cellular differentiation in the human uterus. In an animal model, the reduction or absence of HOXA10 in the uterine endometrium leads to subfertility or infertility due to the inability of the embryo to implant [19]. In a study by Rackow and Taylor, HOXA10 was shown to be significantly reduced in submucosal fibroids compared to controls, and while patients with intramural fibroids had a trend towards lower HOXA10, the trend was not significant [20]. In contrast, Matsusaki and colleagues did demonstrate a significant decrease in HOXA10 in patients with intramural fibroids compared to healthy patient controls [21]. There were key differences in these studies. The former study enrolled fertile patients at time of hysterectomy, and HOXA10 was evaluated in the follicular phase. The latter study compared subfertile patients with fertile controls and measured HOXA10 via endometrial biopsy in the luteal phase, 7 days after luteinizing hormone (LH) surge was detected. HOXA10 is up regulated in the secretory phase, which may play a key role in detecting a change in HOXA10 expression in the latter study. A recent study measured HOXA10 before and after myomectomy. Although underpowered, the study found a nonsignificant trend towards decreased HOXA10 in patients with intramural fibroids before myomectomy compared to after myomectomy [22]. A possible mechanism for this interaction is the secretion of TGF-β3 by leiomyomas [23]. TGF-β3 (transforming growth factor, beta 3) induces resistance of a local growth factor, bone morphogenetic protein 2 (BMP-2), which ultimately leads to suppressed HOXA10, resulting in defective uterine decidualization.

Glycodelin is another implantation factor that has been studied in patients with fibroids. Glycodelin has many properties, including promoting angiogenesis and suppressing natural killer (NK) cells. It also appears to inhibit binding of the spermatozoa to the zone pellucida. Like HOXA10, glycodelin levels are reduced in the follicular phase and increased at time of implantation [24]. Glycodelin has also been shown to have altered expression in patients with uterine fibroids. A prospective cohort study compared uterine flushings obtained 7 days after a LH surge in fertile women with and without submucosal fibroids. Glycodelin and interleukin 10 (IL-10) was significantly reduced in the fibroid cohort [25]. A separate study evaluated glycodelin levels in plasma and uterine flushings in 44 women seeking infertility care. This prospective cohort study obtained plasma and endometrial glycodelin levels in the follicular phase. Women with a normal uterine cavity and absence of fibroids served as controls. Study subjects included submucosal (*n* = 7) and intramural fibroids (*n* = 5), as well as patients with uterine polyps (*n* = 12). The fibroid and polyp cohorts had significantly higher levels of plasma glycodelin than the control group, and only polyps had a significantly elevated uterine cavity glycodelin. Due to the low number of intramural fibroids (*n* = 5), a subgroup analysis was not performed [26]. It is unclear from these two studies whether fibroids increase glycodelin levels in the follicular phase or decrease glycodelin in the secretory phase. A study by Sanoee et al. evaluated endometrial expression of glycodelin in the luteal phase before and after removal of intramural fibroids that did not distort the uterine cavity, and found a significantly lower level of glycodelin after fibroid removal compared to the baseline level before surgery [27]. Twelve infertile patients with intramural fibroids greater than 5 cm not impinging on the endometrial cavity were enrolled in this study.

Leukemia inhibitory factor (LIF) is essential for blastocyst implantation [28], and has also been studied in endometrium affected by uterine fibroids. Endometrial LIF mRNA expression was measured prospectively in patients with submucosal or intramural fibroids, and compared with normal uterine cavity patients serving as controls. Samples were acquired via curettage during the follicular phase. There was no difference noted in LIF mRNA expression between the three cohorts [20]. A similar study was performed evaluating the expression of LIF using quantitative RT-PCR in patients with abnormal uterine cavities (submucosal fibroids and polyps) and control patients. Patients with intramural fibroids were not included. Expression was measured in all phases on the menstrual cycle, and LIF was noted to be significantly reduced in the mid-secretory phase when compared to control [29]. Unfortunately, no comparison of LIF expression has been performed in patients with intramural fibroids during the secretory phase. Other implantation factors including E-cadherin, important for blastocyst attachment and subsequent invasion, and β-catenin, a mediator of the Wnt signaling pathway, have been suggested as potential markers of implantation that are affected by uterine fibroids. No differences in mid-secretory expression between controls and patients with intramural or subserosal fibroids were found in a prospective cohort trial [30].

Horcajades et al. performed a unique study assessing the effect of intramural fibroids on endometrial implantation factors noted to have uterine fibroids while undergoing evaluation for donor oocyte in vitro fertilization cycle (IVF) [31]. In this study, endometrial samples were acquired before patients underwent myomectomy. Fibroids were grouped into less than/greater than 5 cm, and were not encroaching upon the uterine cavity. A microarray was performed on patient’s endometrial tissue samples, and compared to a control group without uterine fibroids also undergoing donor oocyte IVF. The microarray consisted of 25 implantation factor genes, which the authors previously published as the relevant genes of implantation interest. These genes were identified by comparing endometrial tissue sample arrays obtained during the natural cycle window of implantation, an IVF-stimulated cycle during the window of implantation, and endometrium samples obtained during the non-receptive portion of a natural menstrual cycle [32]. Three genes of interest were down-regulated in the fibroid cohort, those being GPx3, placental protein 14 (or glycodelin), and aldehyde dehydrogenase three family, member B2. The authors noted that these genes were also down-regulated in nonoptimal conditions such as controlled ovarian hyperstimulation cycles using GnRH agonists, and in the presence of an intrauterine device. It is worth pointing out that the fibroid cohort in this study did not have a listed history of subfertility or recurrent pregnancy loss. Thus, it is unclear whether infertile patients with intramural fibroids as the only known cause of infertility compared to age matched controls may have resulted in a different outcome. In a recent study, another implantation factor, GP×3 (or glutathione perioxidase 3) has been measured in infertile patients with intramural fibroids before and after myomectomy but the rise in levels after surgery was insignificant [27].

Intramural leiomyomas appear to reduce of HOXA10, and possibly alter glycodelin levels in the secretory phase endometrium. Prior research does not demonstrate changes in leukemia inhibitory factor in the presence of intramural fibroids. A microarray study did not demonstrate differences in select implantation factors between patients with intramural myomas and controls, but it is not clear if the fibroid cohort suffered from subfertility or early pregnancy loss.

### Alterations in the uterine junctional zone

The inner third of the myometrium, the layer that immediately abuts the endometrium, has been labeled as the uterine junctional zone. This layer has been shown to be architecturally different from the rest of the myometrium, and is the origin of myometrial contractions on cine MR(cine MR refers to a cycle of rapidly recorded images taken in sequence and displayed in an active movie display) [33]. Thickening or disruption of this layer by adjacent fibroids may also contribute to poor reproductive performance [34]. The junctional zone changes dramatically in response to estrogen and progesterone with cycle changes, in contrast to the rest of the myometrium. Estrogen and progesterone receptor expression is also cycle phase dependent [35]. Around 5–7 days after ovulation, at the time of implantation, myometrial contractions are limited and decidualization of the endometrium and junctional zone takes place. The differentiation of tissue in the decidualization process takes place in part by the work of uterine natural killer cells (uNK) and macrophages. uNK cells are the most abundant immune cells in the uterus at the time of implantation, and an alteration of uNK cell number has been associated with implantation failure [36].

Kitaya et al. looked at uNK cells and macrophages in patients with and without uterine fibroids [37]. After hysterectomy, endometrium was sampled from the nearest and largest fibroid, and a second sample was obtained from the contralateral side of the uterus. In the mid-secretory phase, uNK cells were significantly reduced, and macrophage cells were significantly increased in endometrium near fibroids compared with endometrium away from the fibroids, and significantly reduced when compared to healthy controls. Unfortunately, the data in this study was not stratified by uterine fibroid type, and although fertile, the average age of participants was over 40 years in both cohorts. Since intramural uterine fibroids often abut the endometrium without distorting the cavity, alterations in adjacent macrophages could alter the decidualization process needed for implantation.

It is also possible that physical disruption of the junctional zone occurs with the presence of intramural uterine fibroids, leading to implantation failure as seen in infertility and early pregnancy loss [38]. There also may be changes in expression of estrogen and progesterone (and their associated receptors) in the junctional zone with increased expression of aromatase, changes in estrogen receptor predominance, and possible changes in progesterone receptor expression that occurs with intramural fibroids [39–41]. More work in this area with a subfertile population (stratified for fibroid type and cavity involvement) is needed before further inferences can be made.

### Uterine myometrial peristalsis

Cine mode magnetic resonance imaging (MRI) has led to the capture of several kinematic events of the human pelvis. One such event is the presence of myometrial contractions of the uterus [42]. Contractions increase in frequency from menses to the mid-ovulatory phase of the cycle, and progress from the cervix to the fundus. After ovulation, the frequency of contractions reduces (from 2–3 times per minute) to relatively quiet at time of implantation. The direction of contractions or peristalsis is also reversed in the luteal phase [43]. An initial study looking at the effects of fibroids on uterine contractions showed contractions present with all fibroid types (SM, IM, SS). Peristalsis was focally obscured in 33 % of SM fibroid patients, and not in other fibroid types. This study was limited by crossover between fibroid types (some SM patients also had IM fibroids), small patient enrollment (22 total), and differing menstrual phases within each fibroid type [44]. Further studies demonstrated increased myometrial peristalsis in patients with IM and SM fibroid types when compared with healthy controls during the mid-luteal cycle phase, and decreased peristalsis in peri-ovulatory phase [45, 46].

The link between infertility and abnormal uterine peristalsis in patients with intramural fibroids was explored by a study by Yoshino and colleagues. Ninety-five infertility patients with IM fibroids only were included in this study. Cine mode MRI was performed during the period of implantation (luteal phase days 5–9), and patients were categorized into low frequency and high frequency peristalsis groups. High frequency was categorized as have greater than or equal to two peristaltic movements within 3 min, and patients were categorized as low frequency if they had less than two movements in 3 min. Patients in both groups had similar numbers and maximum diameter of fibroids (about half in each group had endometrial cavity involvement by fibroids). Patients in both groups were treated with increasing levels of therapy for infertility (natural cycle, ovulation induction with oral agents and/or injectable gonadotropins, and intrauterine insemination). The pregnancy rate over 2 years was 34 % in the low-frequency peristalsis group, and 0 % in the high-frequency peristalsis group, emphasizing that abnormal uterine peristalsis is a likely a cause of infertility, and can result from the presence of intramural fibroids [47]. Limited data suggests that myomectomy and uterine artery embolization may reduce the increased peristalsis seen in some patients with IM fibroids [48, 49]. A retrospective study by Yoshino et al. looked at 15 patients with IM fibroids that had high frequency uterine peristalsis (≥2 peristaltic movements in 3 minutes) in the mid-luteal phase. Following myomectomy, peristalsis normalized in 14 of 15 patients. Pregnancy rate was 40 % over the course of one year after surgery [50].

### The fibroid pseudocapsule

Leiomyomas of the uterus are surrounded by an easily identifiable external layer at the time of myomectomy, referred to as the fibroid pseudocapsule (PC). The PC surrounds the rest of the myoma with a bundle of smooth muscle cells and a vascular capsule, which provides the myoma its blood supply [51]. Research has identified the pseudocapsule as a site rich in neurotransmitters and neurovascularization. Endoglin and CD34, a marker of neovascularization, is upregulated in the PC compared to fibroid and surrounding myometrium, implicating the PC’s role in neovascularization of the fibroid [52, 53]. The capsule thickness varies with fibroid type and location. Submucosal fibroid PCs are significantly thicker than intramural myoma PCs, and intramural PCs are significantly thicker than subserosal PCs. Thickness also increases as the fibroid location is closer to the cervix [54]. Thickness of capsule likely alters expression of numerous modulators, as PC found closer to the cervix have higher expressions on enkephalin and oxytocin. These neuropeptides may alter fertility by inducing abnormal uterine contractions. The capsule and associated cytokines, growth factors and hormones may be responsible for the abnormal uterine peristalsis seen in other myoma research studies, or pregnancy complications seen in fibroid uteri [55]. Intramural fibroid PC has also been associated with increased levels of neurotensin, neuropeptide tyrosine, and protein gene product 9.5- all of which can induce muscular contractions [56]. Premature uterine contractions could lead to disruption of early pregnancies, or could be the cause of preterm delivery as seen in women with large intramural fibroids [57].

## Conclusions

**Fig. 1:**
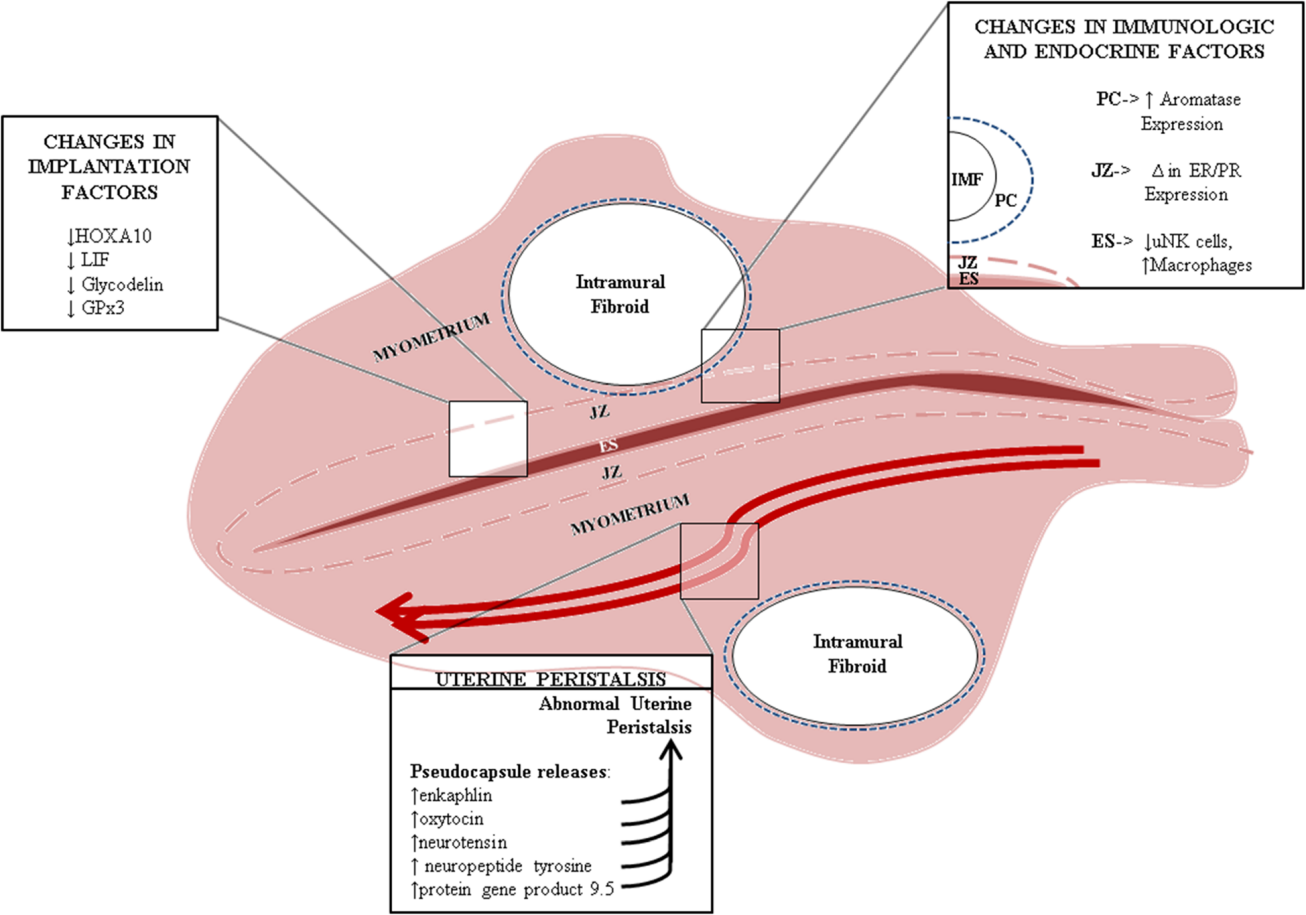
Potential causes of decreased fertility and  pregnancy loss in patients with intramural fibroids. (IMF- intramural fibroid, PC- fibroid pseudocapsule, JZ- uterine junctional zone, ES- endometrium, ER- estrogen receptor, PR- progesterone receptor, uNK- uterine natural killer cells.)

Very little is known about the mechanism by which intramural fibroids impact human fertility (see summary of current known data in Fig. 1). Molecular detection of uterine implantation factors that demonstrated significant findings in patients with submucosal fibroids, often demonstrated a non-significant trend towards similar findings in intramural fibroid patients. More robust study design and higher numbers of patients are needed to maximize the potential of detecting a smaller but important difference. The use of MRI to detect uterine peristalsis is very promising, but further widespread application of this technique is needed to see if both the frequency and impact of this phenomenon with intramural fibroids.

Future studies should be designed comparing infertile women with intramural fibroids as the only cause of infertility, or patients with IM fibroids and recurrent pregnancy loss, to fertile women without uterine fibroids. In addition, implantation factors should likely only be measured in their time of highest expression, during the window of implantation. Further studies are needed after myomectomy, uterine artery embolization, MRI guided high frequency ultrasound therapy, and various medical therapies to provide insight following therapy designed to improve fertility but lacking rigorous proof.
